# Pharmacological interventions for anthracycline-induced cardiotoxicity in breast cancer: a systematic review and meta-analysis of randomized controlled trials

**DOI:** 10.1007/s10549-025-07791-7

**Published:** 2025-08-05

**Authors:** Pinyadapat Vacharanukrauh, Kyle J. Miller, Sheikh M. Alif, Fergal Grace, Muhammad Aziz Rahman

**Affiliations:** 1 Institute of Health and Wellbeing, Federation University Australia, Ballarat, VIC Australia; 2 School of Psychological Sciences, Monash University, Melbourne, VIC Australia; 3 School of Public Health and Preventive Medicine, Monash University, Clayton, VIC Australia; 4 Faculty of Public Health, Universitas Airlangga, Surabaya, Indonesia

**Keywords:** Anthracyclines, Chemotherapy, Cardioprotective interventions, Breast cancer, Randomized clinical trials, Network meta-analysis

## Abstract

**Purpose:**

This study aimed to systematically assess the efficacy of cardioprotective agents in preventing anthracycline-induced cardiotoxicity in patients with breast cancer using a comprehensive network meta-analysis (NMA).

**Methods:**

This study included patients with breast cancer undergoing anthracycline-based chemotherapy. Randomized controlled trials (RCTs) published before March 2020 were identified through systematic searches in MEDLINE, Cochrane CENTRAL, Web of Science, and CINAHL. The primary outcome was left ventricular ejection fraction (LVEF), assessed using cardiac magnetic resonance imaging, multigated radionuclide angiography, or echocardiography. The NMA integrated direct and indirect comparisons to estimate the relative effectiveness of pharmacological interventions.

**Results:**

The systematic review included 31 RCTs with 3,228 participants, whereas the NMA synthesized 25 effect sizes from 15 RCTs. Mineralocorticoid receptor antagonists (MRAs) [standardized mean difference (SMD): −1.78, 95% confidence interval (CI): −2.81 to −0.75] and trimetazidine (SMD: −1.12, 95%CI: −2.32 to −0.09) exhibited the most substantial cardioprotective effects. Dexrazoxane (SMD: −0.53, 95%CI: −1.90 to −0.02) and β-blockers (SMD: −0.34, 95%CI: −0.70 to 0.02) showed potential benefits, albeit with greater uncertainty. Direct comparisons showed that dexrazoxane was more effective than β-blockers (SMD: −1.25, 95%CI: −2.22 to −0.48), with mineralocorticoid receptor antagonists (MRAs) outperforming both. Despite heterogeneity and potential publication bias, mineralocorticoid receptor antagonists (MRAs) and trimetazidine consistently ranked as the most effective interventions. LVEF findings confirmed the cardioprotective benefits of β-blockers, ARBs, ACE inhibitors, and dexrazoxane.

**Conclusions:**

RCT evidence suggested that cardioprotective drugs effectively mitigate anthracycline-induced LVEF decline. However, the lack of direct head-to-head trials limits definitive conclusions on comparative efficacy, warranting trials in patients with lower baseline LVEF to optimize cardioprotective strategies.

**Supplementary Information:**

The online version contains supplementary material available at 10.1007/s10549-025-07791-7.

## Introduction

Cardiotoxicity and heart failure are critical complications of anthracycline-based chemotherapy, significantly increasing morbidity and mortality among cancer patients [1, 2]. The adverse effects of these agents are primarily evaluated using left ventricular ejection fraction (LVEF) and cardiac biomarkers, which constitute indicators of myocardial injury and systolic dysfunction [3]. A reduction in LVEF is widely acknowledged as an early sign of anthracycline-induced cardiotoxicity. However, the long-term impact of these agents on cardiac function remains unclear [4]. Addressing anthracycline-induced cardiotoxicity is a major challenge in breast cancer survivorship, necessitating effective prophylactic strategies, including early detection of subclinical toxicity, modified anthracycline formulations, and cardioprotective pharmacological interventions [5].

Several classes of cardioprotective agents, including β-blockers, angiotensin-converting enzyme inhibitors (ACEIs), angiotensin receptor blockers (ARBs), mineralocorticoid receptor antagonists (MRAs), and dexrazoxane, have been investigated for mitigating anthracycline-induced cardiac toxicity [6, 7]. While previous studies have demonstrated their potential benefits, direct head-to-head comparisons remain lacking, causing difficulty in establishing the most effective intervention [8].

Considering the existing uncertainty, a network meta-analysis (NMA) is imperative for synthesizing direct and indirect evidence to facilitate a robust comparative assessment of available pharmacological interventions. This study aimed to provide a comprehensive and methodologically rigorous evaluation of prophylactic pharmacological strategies for patients with breast cancer undergoing anthracycline-based chemotherapy, thereby supporting evidence-based clinical decision-making. The primary objective was to assess the efficacy of cardioprotective agents in preventing LVEF decline and chemotherapy-induced cardiotoxicity. Furthermore, an NMA enables indirect comparisons between interventions not directly evaluated in randomized trials, enhancing clinical evidence robustness.

## Methods

### Protocol and registration

The network meta-analysis was conducted following the Preferred Reporting Items for Systematic Reviews and Meta-Analyses for Network Meta-Analyses (PRISMA-NMA) guidelines [9] and methodological principles for evaluating multiple treatments, as described in the Cochrane Handbook for Systematic Reviews of Interventions. The review protocol was prospectively registered in the PROSPERO database (CRD42020168609) (https://www.york.ac.uk/crd/) to ensure methodological transparency and rigor.

### Search strategy

A thorough systematic literature search was performed across multiple electronic databases, including MEDLINE (PubMed), Cochrane CENTRAL, Web of Science, and CINAHL, encompassing studies published from the inception of each database up to June 15, 2022. Advanced text mining techniques were applied to optimize the selection of search terms regarding the target population (breast cancer), intervention (cardioprotective agents), and study design (randomized controlled trials). Specifically, Python’s Natural Language Toolkit (NLTK) was used for text processing, whereas term frequency-inverse document frequency (TF-IDF) and word embedding models (such as Word2Vec and GloVe) facilitated the identification of relevant keywords. The final search strategy incorporated both MeSH and free-text terms, systematically combined using Boolean operators to enhance retrieval precision. The search and screening process was independently conducted by two reviewers, with any discrepancies resolved through discussion with a third reviewer. A comprehensive list of search terms and a representative search strategy are presented in Table 1.

**Table 1:** PubMed search strategy

((((((((((((((((RCT[Title/Abstract]) OR Experimental study[Title/Abstract]) OR Randomis*[Title/Abstract]) OR randomized control*[Title/Abstract]) OR randomised[Title/Abstract]) OR randomized*[Title/Abstract]) OR randomised control trial[Title/Abstract]) OR Randomised clinical[Title/Abstract]) OR Randomized clinical[Title/Abstract])) AND ( "0001/01/01"[PDat]: "2022/06/15"[PDat]) AND Humans[Mesh] AND English[lang])) AND Humans[Mesh] AND English[lang])) AND ((((((breast cancer[MeSH Terms] AND ( "0001/01/01"[PDat]: "2022/06/15"[PDat]) AND Humans[Mesh] AND English[lang]) AND Humans[Mesh] AND English[lang])) AND (((((((((((((Adrenergic beta-Antagonists) OR Angiotensin-Converting Enzyme Inhibitors) OR Angiotensin Receptor Antagonists) OR Diuretics) AND ( "0001/01/01"[PDat]: "2022/06/15"[PDat]) AND Humans[Mesh] AND English[lang])) OR (((((((Cardioxane) OR Cardioxan) OR Dexrazoxan*) OR Zinecard) OR Razoxane) OR Dexrazoxane) AND ( "0001/01/01"[PDat]: "2022/06/15"[PDat]) AND Humans[Mesh] AND English[lang])) AND ( "0001/01/01"[PDat]: "2022/06/15"[PDat]) AND Humans[Mesh] AND English[lang])) AND ( "0001/01/01"[PDat]: "2022/06/15"[PDat]) AND Humans[Mesh] AND English[lang]))) AND Humans[Mesh] AND English[lang])) AND ((((((((((((anthracycline) OR aclarubicin) OR daunorubicin) OR daunomycin) OR carubicin) OR plicamycin) OR doxorubicin) OR epirubicin) OR idarubicin) OR nogalamycin) OR menogaril) AND ( "0001/01/01"[PDat]: "2022/06/15"[PDat]) AND Humans[Mesh] AND English[lang])) AND Humans[Mesh] AND English[lang])) AND Humans[Mesh] AND English[lang])	

### Inclusion criteria

Studies were eligible for the NMA if they met the following criteria: (1) utilized a randomized controlled trial (RCT) design; (2) included female participants aged ≥ 18 years; (3) included patients with confirmed breast cancer diagnosis; (4) administered anthracycline-based chemotherapy as part of primary tumor treatment; (5) optionally included concomitant radiation therapy; (6) evaluated a single cardioprotective pharmacological agent, such as ACEIs, ARBs, antimitotics, β−1 adrenergic receptor blockers, trimetazidine, or MRAs; (7) assessed LVEF as the primary outcome, alongside secondary measures such as cardiac biomarkers, documented cardiac events (e.g., heart failure), treatment-related adverse events, and cancer-related mortality; (8) published in a peer-reviewed, English-language medical journal; and (9) trial completion before the COVID-19 pandemic lockdown (March 22, 2020).

### Data collection and quality assessment

The study selection and data extraction process were conducted independently by two investigators (PV and KJM) using a standardized data collection form. During initial screening, the titles and abstracts of all retrieved articles were assessed according to predefined inclusion and exclusion criteria. Studies meeting these criteria underwent a full-text review for further evaluation. Any discrepancies were resolved through discussion and consensus with a third investigator (FG). When necessary, the corresponding authors of the included RCTs were contacted—at most twice—to obtain additional study details.

A standardized Microsoft Excel data extraction form was employed to systematically collect key study information, including study identification details, publication year, country, application of the intention-to-treat principle, patient demographics, cardioprotective agents, intervention details (prior and concurrent therapies), treatment context (metastatic or adjuvant setting), cardiotoxicity management, chemotherapy regimens, anthracycline type, cumulative anthracycline dose, history of prior anthracycline exposure, sample size, mean patient age, treatment duration, follow-up period, comorbidities, radiotherapy specifics (frequency, dose, cumulative exposure), and risk of bias.

Each included study’s risk of bias was evaluated using the Cochrane Collaboration’s risk of bias assessment tool, examining seven key domains: random sequence generation, allocation concealment, participant and study personnel blinding, outcome assessment blinding, outcome data completeness, selective outcome reporting, and other potential sources of bias. Two investigators (PV and KJM) independently conducted the evaluation, and any discrepancies were resolved through discussion with a third investigator (FG).

### Primary and secondary outcomes

The primary outcome was the change in LVEF from baseline to follow-up in patients receiving cardioprotective interventions relative to those in the control group. Mean LVEF changes were determined using the reported mean (M), standard deviation (SD), and sample size (n). Effect sizes were calculated or converted into standardized mean differences (SMD) using pooled standard deviations or established transformation methods, including Hedges’ g correction to adjust for small-sample bias. If essential statistical data were unavailable, alternative estimations were applied where feasible; otherwise, studies were omitted from the quantitative analysis.

Secondary outcomes included the incidence of chemotherapy-induced cardiotoxicity, cancer-related mortality, and safety outcomes, such as serious adverse events, treatment-related adverse effects, and known toxicities associated with anthracycline therapy.

The NMA framework operates under the assumption that effect estimates are derived from a collection of RCTs with comparable effect modifiers [10]. However, the transitivity assumption may be violated if the distribution of these effect modifiers substantially differs among studies. To address this potential issue, comparator groups were categorized based on the type of cardioprotective agents, including ACEIs, ARBs, antimitotics, β−1 blockers, trimetazidine, and MRAs.

### Statistical analysis

Data analysis was conducted using Stata software (Stata Statistical Software: Release 15, StataCorp LLC, College Station, TX) to perform a multivariate random-effects meta-analysis [11]. The change in LVEF from baseline to post-chemotherapy was treated as a continuous variable, reported as the SMD with corresponding 95% confidence intervals (CI). Hedges’ g correction was applied to estimate SMD values, and effect sizes were interpreted based on Cohen’s criteria, where 0.2 represents a small, 0.5 a medium, and 0.8 a large effect [12].

The degree of heterogeneity was evaluated using the I^2^ statistic, classified as follows: low (0–25%), moderate (25–50%), substantial (50–75%), and high (> 75%). Cochran’s Q test was applied to assess the statistical significance, with a threshold of p < 0.10. Between-study heterogeneity and inconsistency were examined in the NMA using global inconsistency models based on the design-by-treatment interaction approach and local inconsistency tests employing node-splitting analysis. A random-effects model using either a Bayesian hierarchical framework or the frequentist DerSimonian and Laird method was employed to account for between-study variability. The consistency of direct and indirect evidence was examined by comparing estimates from both sources. Sensitivity analyses were conducted by excluding studies with a high risk of bias or exhibiting extreme effect sizes.

Publication bias and small-study effects were examined through comparison-adjusted funnel plots and Egger’s test. The certainty of the evidence supporting the network estimates was assessed using the Grading of Recommendations Assessment, Development, and Evaluation (GRADE) framework. Statistical significance was set at a two-sided p-value < 0.05 [12].

A network plot was generated using the ‘network plot’ command to display direct treatment comparisons. The contribution plot (‘net weight’) was utilized to demonstrate the relative influence of direct and indirect comparisons within the network model, whereas the interval plot (‘interval plot’) was used to visualize ranking uncertainty. Treatment efficacy was evaluated using the surface under the cumulative ranking curve (SUCRA) and mean ranks, with analyses performed using the ‘sucra’ and ‘network rank’ commands. Additionally, inconsistency was examined through the ‘ifplot’ command and inconsistency models, which assessed loop-specific and global consistency. Publication bias and small-study effects were examined through comparison-adjusted funnel plots using the ‘netfunnel’ command.

### Data synthesis

A systematic approach was employed to integrate findings across studies for primary and secondary outcomes. Pairwise meta-analyses were conducted for direct comparisons using a random-effects model to account for inter-study variability, whereas an NMA was conducted for indirect comparisons using either a Bayesian hierarchical model or a frequentist approach, ensuring coherence between direct and indirect estimates.

Missing or inconsistent data were managed through multiple imputation techniques when applicable. Sensitivity analyses were conducted to evaluate the potential influence of missing data on the overall findings. If studies reported incomplete outcome measures, effect sizes were estimated using standard conversion methods based on the available data. The analysis adhered to the assumption of consistency, and any deviations were explored through node-splitting and inconsistency models. This rigorous methodological framework ensured a comprehensive and reliable synthesis of evidence across the network.

## Results

**Fig. 1:**
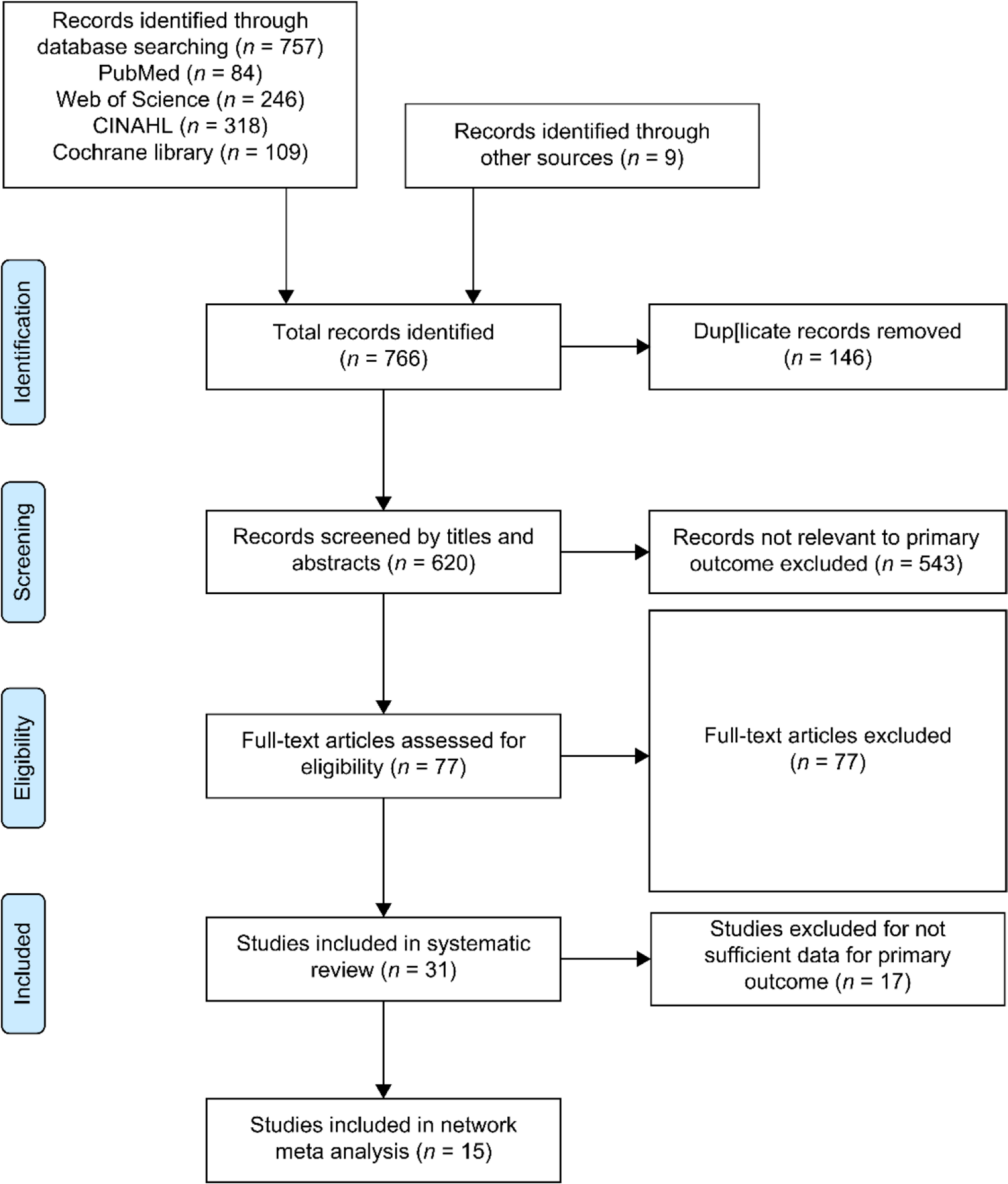
Flowchart of the study selection process

The systematic literature search identified 766 potentially relevant records. After removing duplicates (*n* = 146) (Fig. 1), the remaining RCTs underwent title and abstract screening by two independent reviewers, achieving a 96% agreement rate. After the initial screening, 77 full-text articles were evaluated for eligibility, of which 46 studies were excluded based on predefined criteria (Fig. 1). The full-text review was independently conducted by two researchers, achieving a 95% agreement rate. The systematic review encompassed 31 RCTs with 3,228 participants. Among these, 15 RCTs (*n* = 1,545 participants, 25 effect sizes) met the inclusion criteria for the NMA.

**Fig. 2:**
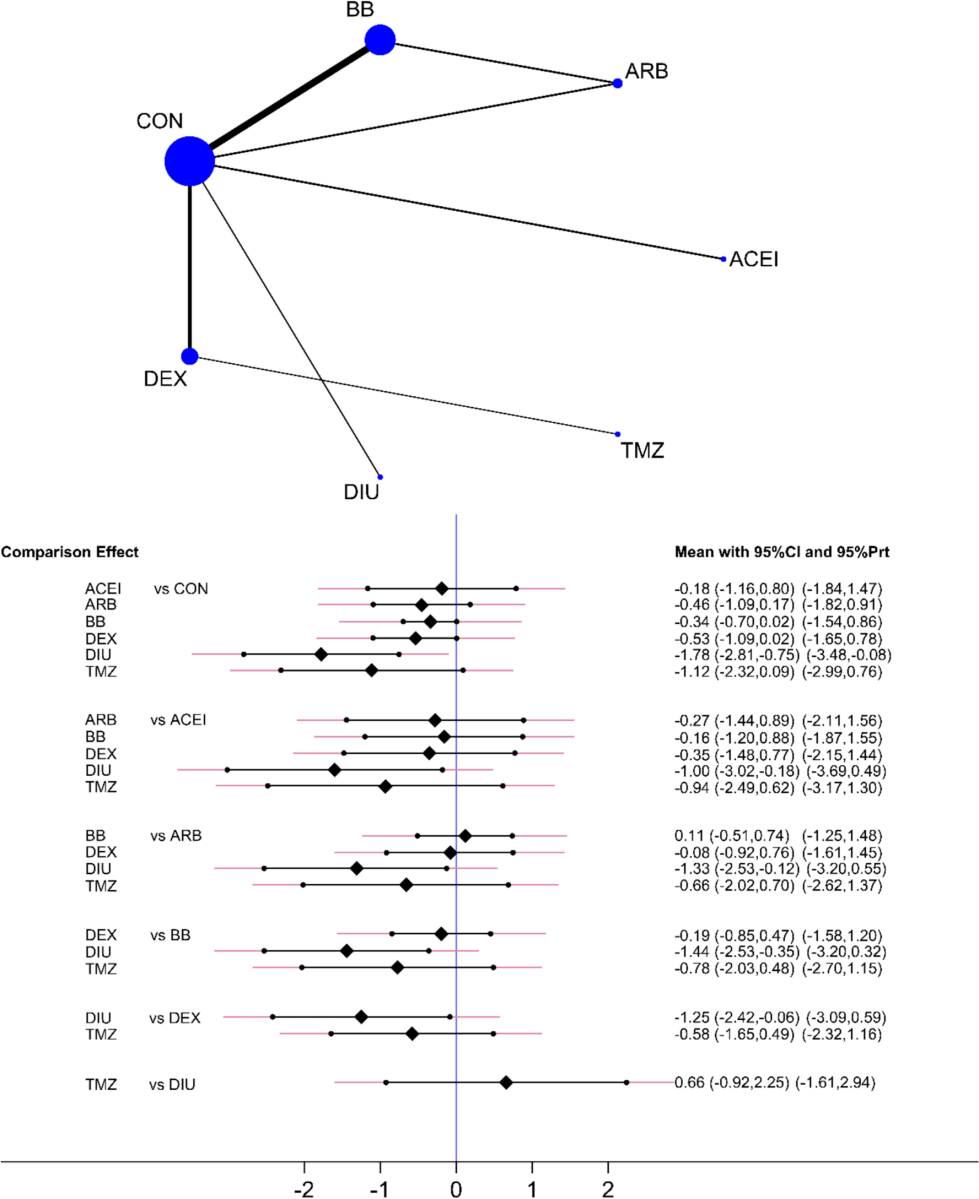
Network plot of included RCTs and predictive interval plot for cardioprotective agents. Black diamonds indicate the estimated effect size (Hedges’ g), with narrower horizontal lines representing confidence intervals (CI) and wider lines depicting predictive intervals (PrI). The blue vertical line marks the null hypothesis (Hedges’ g = 0), while negative values indicate a stronger cardioprotective effect in the comparator (left) group. Abbreviations: *ACEI* angiotensin-converting enzyme inhibitors, *ARB* angiotensin II receptor blockers, BB β−1 adrenergic receptor blockers, *MRA* mineralocorticoid receptor antagonists, *DEX* dexrazoxane, *TMZ* trimetazidine, *CON* control

The NMA encompassed 15 RCTs with 1,545 participants, including 892 in the treatment group and 653 in the control group (Fig. 2). All participants were female patients diagnosed with breast cancer who underwent anthracycline-based chemotherapy, receiving either doxorubicin or epirubicin. The RCTs investigated anthracyclines administered alone or combined with other pharmacological agents and/or radiotherapy. The cumulative anthracycline dose varied across studies, ranging from 160.8 to 960 mg/m^2^. The included studies evaluated various prophylactic cardioprotective agents, including β−1 blockers (8 RCTs), ARBs (2), ACEIs (1), MRAs (1), trimetazidine (1), and dexrazoxane (3) (Table 2).

**Table 2:** Characteristics of participants, interventions, and methodologies in randomized controlled trials included in the systematic review

Author (year)	Treatment M_age_ (SD)	Control M_age_ (SD),	Intervention sample size (n)	Control sample size (n)	Anthracycline cumulative drug dose (mg/m^2^) *Treatment. Control*		Type of protective drug intervention	Intervention Dose mg/day (Dex: mg/m^2^)	Length of intervention (weeks)	Outcomes measure(s)
Akpek (2015) [13]	50(10.8)	50.6(10.1)	43	40	X	X	Spironolactone	25	24	Transthoracic echocardiography LVEF/Cardiac and oxidative biomarkers/Troponin-I
Avila (2018) [14, 15]	50.8(0.1)	52.9(9.05)	96	96	240	240	Beta-blocker (carvedilol)	5	24	A 10% LVEF decline at six months, with elevated troponin I, increased brain natriuretic peptide (BNP), and diastolic dysfunction
Cochera (2018) [16]	53(13)	52 (11)	30	30	521	519	Beta-blocker (nebivolol)	50	24	Echocardiography, LVEF; tissue Doppler and speckle tracking echocardiographic imaging; left ventricular systolic and diastolic function; strain
Davis (2019) [17]	53.9(2)	49.1(12.8)	22	22	X	X	Spironolactone (eplerenone)	12.5	24	Transthoracic echocardiograms, LVEF, systolic and diastolic function; cardiac biomarkers
Elitok (2014) [18]	54.3(9.3)	52.9(11.2)	40	40	535.6	523.3	Beta-blocker (carvedilol)	25	24	Cardiac evaluation, a standard ECG, a conventional echocardiography, and SI. LVEF; fractional shortening; M-mode echocardiography; strain imaging
Gulati (2016) [19]	50.0	X	30	X	X	X	Candesartan–metoprolol	32/100	X	Cardiac MRI, blood samples, physical examinations, and electrocardiograms/LVEF/Cardiac troponin I
	51.7(10.7)	50.8(9.2)	32	32	297.5	301.3	Candesartan	32	X	
	50.5(9.1)		32	32	301.3	301.3	Metoprolol	100	X	
Gulati (2017) [20]	51* (42.0, 59.0)	X	27	X	400	X	Metoprolol with placebo (*n* = 6), candesartan with placebo (*n* = 7), metoprolol with candesartan (*n* = 7), and placebo with placebo (*n* = 7)			Cardiovascular magnetic resonance imaging, echocardiographic assessments, and circulating biomarker levels
	48.5* (43.8, 58.0)	X	94	X	< 400	X	Metoprolol and placebo (*n* = 23), candesartan and placebo (*n* = 25), metoprolol and candesartan (*n* = 23), placebo and placebo (*n* = 23)			
Heck (2018) [21]	49.8	50.3(9.6)		18	160.8*(160.8,241.2)		Candesartan–metoprolol		13* (12.0–16.5)	Cardiovascular magnetic resonance
	52.6(10.2)		20		160.8*(160.8,241.2)	201.0*(160.8,268.0)	Candesartan	32		
	49.2(8.1)		13		160.8*(160.8,201.0)		Metoprolol	100		
Kaya (2013) [22]	51.4(9.4)	50.5(11.1)	27	18	361	348	Beta-blocker (nebivolol)	5	24	Echocardiography (echocardiographic variables) and NT-pro-BNP levels
Lee (2021) [23]	47.8(8.7)	48.5(10.4)	82	43	240 (240.0– 240.0)	240 (240.0– 240.0)	Candesartan	16	24	Transthoracic echocardiogram
	46.6(7.6)	48.5(10.4)	70	43	240 (240.0– 240.0)	240 (240.0– 240.0)	Carvedilol	12.5		
Marty (2006) [24]	50*(31–76)	52* (30–71)	85	79	669(247–936)	608(244–900)	Dexrazoxane	500	24	Cardiac assessment included a physical examination, MUGA scan, or echocardiography, along with evaluations of cardiac event incidence, congestive heart failure occurrence, and tumor response rate
Mross (1993) [25]	53*(32–66)	51*(31–67)	26	25	X	X	Verapamil	480	8	Response rate and overall survival
Nabati (2017) [26]	47.57(8.75)	47.1(12.17)	46	45	359.91	348.56	Beta-blocker (carvedilol)	3.125 mg orally twice a day > > 6.125 mg twice daily	24	Echocardiographic evaluation of LVEF, end-diastolic, and end-systolic volumes
Słowik (2020) [27]	237.5	240	48	48	DOX 237,5 (12,2), EPI 383,3 (102,32)	DOX 240 (0.0), EPI356.2 (166.30)	Ramipril	10	24	Echocardiography, LVEF changes, and serum NT-proBNP levels
Speyer (1988) [28]	57*(29–71)	58.3*(32–76)	47	45	471.3	376.5	Dexrazoxane	1000	9.9 cycles(intervention), 8.1 cycles (control)	Radionuclide cardiac scan/nuclear scan/endometrial biopsy
Speyer (1990) [29]	X	X	65	70	300–1400	300–899	Dexrazoxane	1000	X	Clinical exam/MUGA (serial resting and exercise gated pool at baseline and fixed time point)
Speyer (1992) [30]	55.5	56.2	76	74	558	407.4	Dexrazoxane	1000	12.3cycles(intervention), 8.8cycles (control)	A clinical score for chronic heart failure to these events and analysis of MUGA scans/LVEF
Sun (2015) [31]	53.47(5.45)	55.11(2.36)	40	40	480	480	Dexrazoxane	800	18	Echocardiographic assessment of systolic and diastolic function using conventional methods and tissue Doppler imaging ECG
Sun (2016) [32]	53.8(4.99)	55.25(3.75)	51	52	480	480	Dexrazoxane	800	18	Standard ECG and 24-h Holter monitoring, analyzing low-frequency (LF, 0.04–0.15 Hz), high-frequency (HF, 0.15–0.40 Hz), and the LF/HF ratio to assess autonomic balance
Swain (1997) [33]	57*(33–81)	56*(25–77)	102	99	≥ 300	≥ 300	Dexrazoxane	500–1000	≥ 7 cycles	Ejection fractions were evaluated using MUGA scans. A cardiac event was defined as the onset of congestive heart failure or a decline in left ventricular ejection fraction exceeding 20 percentage points from baseline
Swain (1997) [33, 34] study 1: 088001	58*(26–84)	56*(25–82)	168	181	≥ 300	≥ 300	Dexrazoxane	1000	18	Cardiac evaluation included a baseline physical examination, electrocardiography, and assessment of resting left ventricular ejection fraction via multigated acquisition nuclear scanning
study2: 088006	56*(35–76)	59 5*(23–79)	81	104	≥ 300	≥ 300		500		
Tallarico (2003) [35]	55.87(12.4)	X	36	X	DOX 180 mg/mL, EPI 960 mg/m	x	Dexrazoxane and TMZ	TMZ, 60 DEX 100 mg/m^2^(IV)		Doppler echocardiography is utilized to evaluate the diastolic function by measuring key parameters such as E-wave velocity, A-wave velocity, and isovolumetric relaxation time
	61.86(10.09)		38				TMZ	60		
	47.25(12.31)		38				Dexrazoxane	DEX 100 mg/m^2^(IV)		
Tashakori (2016) [36]	42*(29–54)	39.9*(29–54)	45	45	240	240	Carvedilol (non-selective beta-blocker)	6.25 mg twice daily		Cardiac Monitoring and ECG
Venturini (1996) [37]	57*(32–73)	57*(34–74)	84	78	702	713	Dexrazoxane	500		Cardiac assessment (ECG, chest x-rays, and MUGA scan
Vici (1998) [38]	55*(26–71)	58*(33–72)	45	50	960	960	Dexrazoxane	1000		Cardiac monitoring (MUGA Scan and RIS), LVEF/Survival rate

Data are presented as mean ± standard deviation unless stated otherwise, with * indicating median (interquartile range)

*SD* standard deviation, *ECG* electrocardiogram, *MUGA* multiple-gated acquisition, *RNCS* radionuclide cardiac scan, *MRI* magnetic resonance imaging, *LVEF* left ventricular ejection fraction, *BNP* brain natriuretic peptide, *X* data not available, *TMZ* trimetazidine

### Risk of bias

**Fig. 3:**
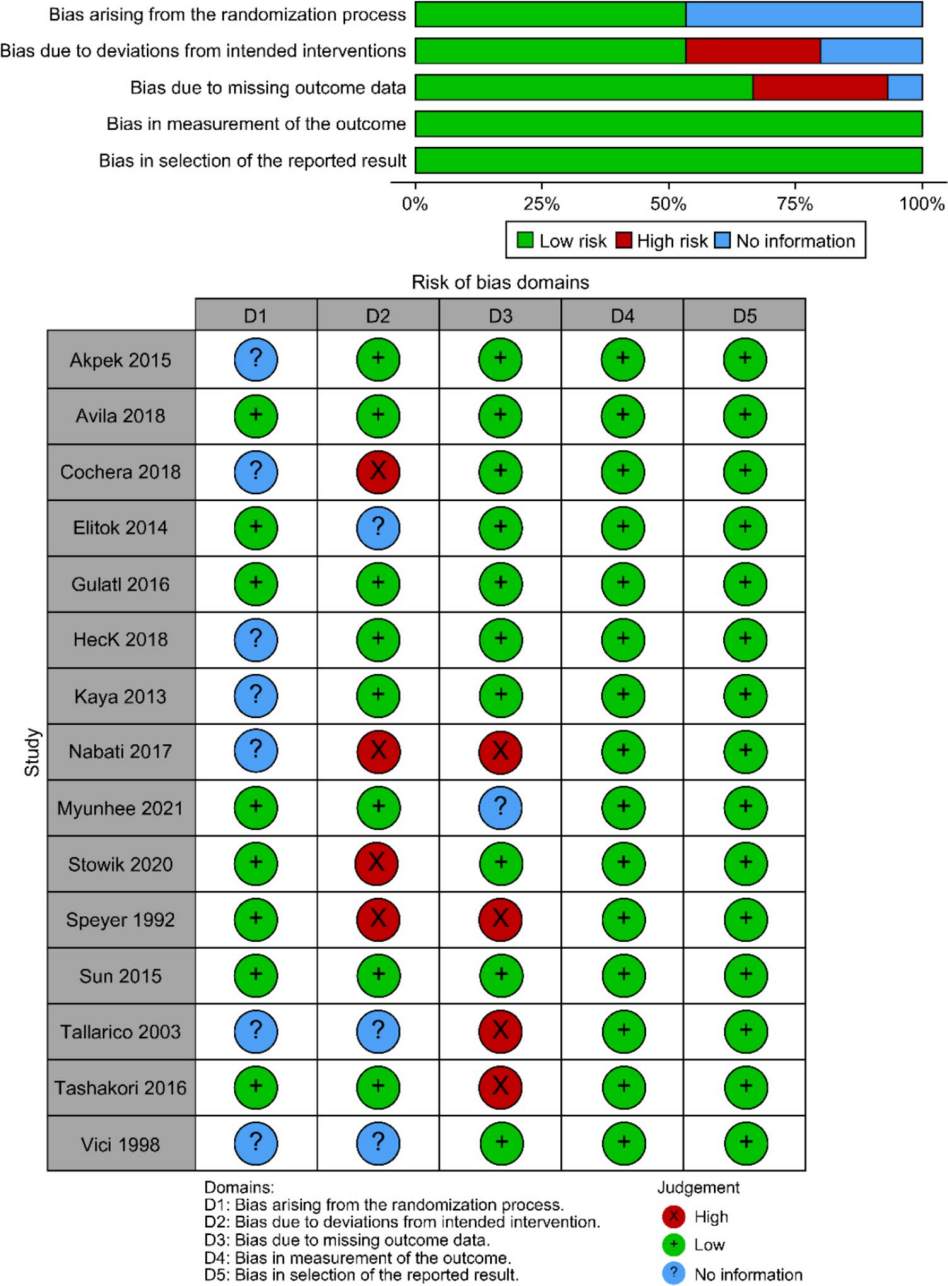
Risk of bias evaluation for randomized controlled trials included in the quantitative analysis

Overall, the RCTs demonstrated a low risk of selective reporting bias and other potential sources of bias. Most studies demonstrated a low overall risk of bias, with only two reporting a moderate risk across three of the seven assessed domains [26, 30]. One notable limitation was the insufficient reporting of outcome assessment blinding (detection bias) in most studies. Moreover, no RCT explicitly described the process of allocation concealment (selection bias), resulting in uncertainty regarding the possible impact of this bias. A detailed assessment of the risk of bias for each study is provided in Fig. 3.

### Results of individual studies and synthesis

Figure 2 presents the complete NMA results. Direct and indirect comparisons were integrated to assess the comparative effectiveness of cardioprotective agents. The network plot highlighted that control and β-blockers were the most frequently investigated interventions. Mineralocorticoid receptor antagonists (MRAs) (−1.78, 95%CI: −2.81 to −0.75) and trimetazidine (−1.12, 95%CI: −2.32 to −0.09) exhibited the most pronounced cardioprotective effects relative to the control group. Dexrazoxane (−0.53, 95%CI: −1.90 to −0.02) and β-blockers (−0.34, 95%CI: −0.70 to 0.02) showed potential benefits; however, their wide credible intervals indicated greater uncertainty in effect estimates. Direct comparisons indicated that dexrazoxane demonstrated significantly greater efficacy than β-blockers (SMD: −1.25, 95%CI: −2.22 to −0.48). Additionally, MRAs were found to be more effective than both β-blockers and ACEIs.

However, the wide predictive intervals in several comparisons suggest substantial between-study heterogeneity, necessitating careful interpretation of findings. Indirect comparisons showed no statistically significant differences between ARBs and β-blockers (0.11, 95%CI:−0.51 to 0.74), whereas MRAs appeared more effective than dexrazoxane (−1.25, 95%CI: −2.42 to −0.08). Additionally, the comparison-adjusted funnel plot indicated potential publication bias, suggesting that smaller or negative studies may be underrepresented in the literature.

Table 3 presents a summary of the changes in LVEF across RCTs included in this NMA (primary outcome), evaluated using echocardiography, cardiac magnetic resonance imaging (MRI), and multigated radionuclide angiography (MUGA).

**Table 3:** Primary outcomes: left ventricular ejection fraction in randomized controlled trials incorporated in the network meta-analysis

Author (year)	Treatment Group	Pre-Treatment Mean	Pre-Treatment SD	Post-Treatment Mean	Post-Treatment SD
**Echocardiography**		LVEF	LVEF	LVEF	LVEF
Akpek (2015) [13]** (Transthoracic)**	MRAs	67	6.1	65.7	7.4
	Control	67.7	6.3	53.6	6.8
Avila (2018) [14, 15] **(Transthoracic)**	Beta-blocker	64.8	4.7	63.9	3.8
	Control	65.2	3.6	63.9	5.2
Cochera (2018) [16]	Beta-blocker	62	4	61	3
	Control	61	2	60	3
Elitok (2014) [16]	Beta-blocker	66	6.1	64.1	5.1
	Control	65	4.5	63.3	4.8
Kaya (2013) [22]	Beta-blocker	65.6	4.8	63.8	3.9
	Control	66.6	5.5	57.5	5.6
Lee (2021) [23]	ARB	64.5	(63.7,65.2) *	63.4	(61.1–65.9)
	Beta-blocker	65.0	(63.0–66.1) *	62.9	(61.0–64.0) *
	Control	64.0	62.8–66.7*	60.2	58.4–63.0*
Nabati (2017) [26]	Beta-blocker	58.7	4.7	57.4	7.52
	Control	61.13	4.9	51.7	6.0
Slowik (2020) [27]	ACEI	66.5	3.8	63.9	3.8
	Control	67.8	3.8	64.5	3.8
Sun (2015) [31]	Dexrazoxane	63	18	64	16
	Control	65	8	65	13
Tallarico (2003) [35]	TMZ	63.6	11.2	61.3	11.6
	Dexrazoxane	60.5	9.3	50.8	13.6
Tashakori (2016) [36]	Beta-blocker	61.3	3.2	61.1	3.4
	Control	59.4	4.2	59.3	4.3
**Cardiac MRI**					
Gulati (2016) [19] Heck (2018) [21]	ARB	62.5	4.4	61.6	4.3
	Beta-blocker	63.3	4.3	60.8	4.4
	Control	63.1	4.4	60.3	4.7
**MUGA scan**					
Vici (1998) [38]	Dexrazoxane			63.4	9.6
	Control			58.1	8.6

Data are reported as mean ± standard deviation unless otherwise specified. * = median (interquartile range)

*SD* standard deviation, *MUGA* multiple-gated acquisition, *MRI* magnetic resonance imaging, *LVEF* left ventricular ejection fraction

The included studies reported varying degrees of LVEF preservation among different cardioprotective agents, including β-blockers (e.g., carvedilol, nebivolol), ARBs (e.g., candesartan), ACEIs, MRAs (e.g., spironolactone), and dexrazoxane. β-blockers and ARBs were associated with significant cardioprotective effects, as observed in studies by Avila et al. [14, 15], Kaya et al. [22], and Lee et al. [23]. Similarly, spironolactone [13] and ACEIs [27] showed protective effects by attenuating LVEF decline compared to controls. Dexrazoxane, evaluated in multiple trials [31, 35, 38], exhibited robust protective effects, effectively preventing substantial LVEF reductions.

The PRADA trial [19, 21] further reinforced the role of ARBs and β-blockers in preserving cardiac function. Collectively, these findings highlight the efficacy of cardioprotective agents in reducing chemotherapy-induced cardiotoxicity, underscoring the clinical significance of prophylactic interventions in high-risk patients.

### Secondary outcomes

Secondary outcomes of this NMA included adverse events associated with cardioprotective interventions, functional cardiac outcomes, and epidemiological endpoints (Table 4). Adverse event analysis showed that ACEIs, ARBs, β-blockers, and dexrazoxane were generally well tolerated. However, dexrazoxane was associated with hematologic toxicity, including neutropenia and myelosuppression, whereas ACEIs frequently caused hypotension and dry cough. Functional cardiac assessments indicated that β-blockers and ARBs effectively preserved hemodynamic stability and autonomic function, as reflected in improved heart rate, blood pressure, and electrocardiographic parameters. Epidemiological outcomes further highlighted the cardioprotective benefits of dexrazoxane, including significantly reduced heart failure incidence and cardiac adverse events with improved overall survival.

**Table 4:** Secondary outcomes of randomized controlled trials incorporated in the network meta-analysis

**a**. Reported Adverse Events in the Included Studies		
**Treatment group**	**Study**	**Adverse event(s)**
**ACEI**	Mross (1993) [25]	Hair loss, moderate nausea/vomiting, stomatitis/mucositis, hypotension
	Słowik (2020) [27]	Dry cough
**ARB**	Lee (2021) [23]	GI trouble, Dizziness, palpitation, Symptomatic hypotension
**BB**	Avila (2018) [14, 15]	Dizziness, Symptomatic hypotension
	Kaya (2013) [22]	Hypotension and bradycardia
	Nabati (2017) [26]	Hypotension
	Lee (2021) [23]	GI trouble, Symptomatic hypotension
**DEX**	Marty (2006) [24]	Alopecia, nausea, neutropenia, vomiting, leukopenia, anemia, and febrile neutropenia
	Speyer (1988) [28]	Decreased WBC/cell count/platelet/hematocrit, and stomatitis/n/v/infection/alopecia (Myelosuppression slightly more in the intervention group)
	Speyer (1990) [29]	Hematologic toxicity (Neutropenia)
	Speyer (1992) [30]	Alopecia, n/v, stomatitis, Hematologic toxicity (Decreased PLT/and WBC)
	Venturini (1996) [37]	mild hematologic toxicity
	Vici (1998) [38]	Neutropenic fever/Cardiac outcomes/N/V

**b** Functional Outcomes as Secondary Outcomes in Studies Included in the Meta-Analysis																					
Author (year)		HR				SBP				DBP				ECG (RHR)				ECG (LF/HF)			
		Before		After		Before		After		Before		After		Before		After		before	After	Before	After
Akpek (2015) [13]	intervention	75	10	76	13	113	14	113	13	75	8	76	7								
	Control	75	11	77	10	111	15	112	15	75	8	75	8								
Avila (2018) [14, 15]	intervention	80	14.1	78	0	120.3	16.6	110	0	77.9	11.9	71	0								
	Control	82.4	12.6	86	0	124.8	17.2	120	0	78.4	10.2	75	0								
Sun (2016) [32]	intervention					131.6	5.45	129.7	5.1	79.4	5.6	79.6	5.3	77.9	14.4	84.4	12.2	2.1	0.4	2.6	0.4
	Control					128.8	6.25	130.0	5.4	81.2	5.85	81.3	6.1	75.0	12.6	92.5	14.6	2.0	0.3	3.3	1.2

**c** Secondary Outcomes Reported in the Included Meta-Analysis Studies											
Author(s)	Completed intervention size	Completed control size	All sample (after)	All sample (before)	Cardiac adverse events%		HF (%)		The median survival times (months)		A survival analysis (hazard ratio)
					Intervention	Control	Intervention	Control	Intervention	Control	
Marty et al. (2006) [24]	36	43	79	164	13% (95% CI 6% to 22%)	39%, (95% CI 28% to 51%)	1%, (95% CI 0.032% to 7%)	11%, (95% CI 5% to 20%)	13.5 (95% CI 0.2 to 27.8 +)	16.0 (95% CI 0.5 to 25.3)	
Mross et al. (1993) [25]	26	25	51	51					8.9 (95% CI 7.5 to 10.5)	7.4 (95% CI 5.9 to 12.6)	
Speyer et al. (1990) [29]	65	70	135	135	6%	47%					
Speyer et al. (1992) [30]			0	150	8%	50%			18.3	16.7	
Swain (1997) [33, 34]	102	99	201	211	25%	60%	3%	22%			2.2 (95% CI, 1.4 to 3.4)
Swain (1997) [33, 34]			416	534							
Study 1: 088001	141	152	293		15%	31%	0%	8%			1.02 (95% CI, 0.80 to 1.31)
Study 2: 088006	54	69	123		14%	31%	2%	7%			0.82 (95% CI. 0.59 to 1.14)
Venturini (1996) [37]	82	78	160	162			2.40%	5.10%			

*ACEI* angiotensin-converting enzyme inhibitors, *ARB* angiotensin II receptor blockers, *BB* β−1 adrenergic receptor blockers, DEX dexrazoxane, *HR* heart rate, *SBP* systolic blood pressure, *DBP* diastolic blood pressure, *ECG* electrocardiography

### Assessment of inconsistency

Inconsistency network models were applied to assess the agreement between direct and indirect estimates in pairwise and multi-arm comparisons. The assumption of overall consistency was upheld for all treatments (*p* > 0.05), except for MRAs, which displayed significant inconsistency (*p* < 0.01). The comparison of direct and indirect estimates for ARBs versus control and ARBs versus β-blockers demonstrated no significant inconsistency (*p* > 0.05), indicating that indirect estimates were consistent with direct evidence. However, owing to limited data availability, direct–indirect comparisons were not feasible for other treatment comparisons and were instead considered within the GRADE assessment framework.

Loop-specific heterogeneity was assessed using an inconsistency plot. Inconsistency factors (IFs) were reported exclusively for the ARB-β-blockers-Control loop, yielding a calculated IF of 0.29, suggesting no significant inconsistency. Data for assessment in other loops was insufficient, and the consistency assumption was maintained and incorporated into the GRADE evaluation. Comprehensive results from the inconsistency analyses and corresponding plots are presented in Supplementary Fig. S1a, whereas contribution plots depicting the network of cardioprotective effects are shown in Supplementary Fig. S1b.

### Assessment of publication bias and sensitivity analyses

**Fig.4:**
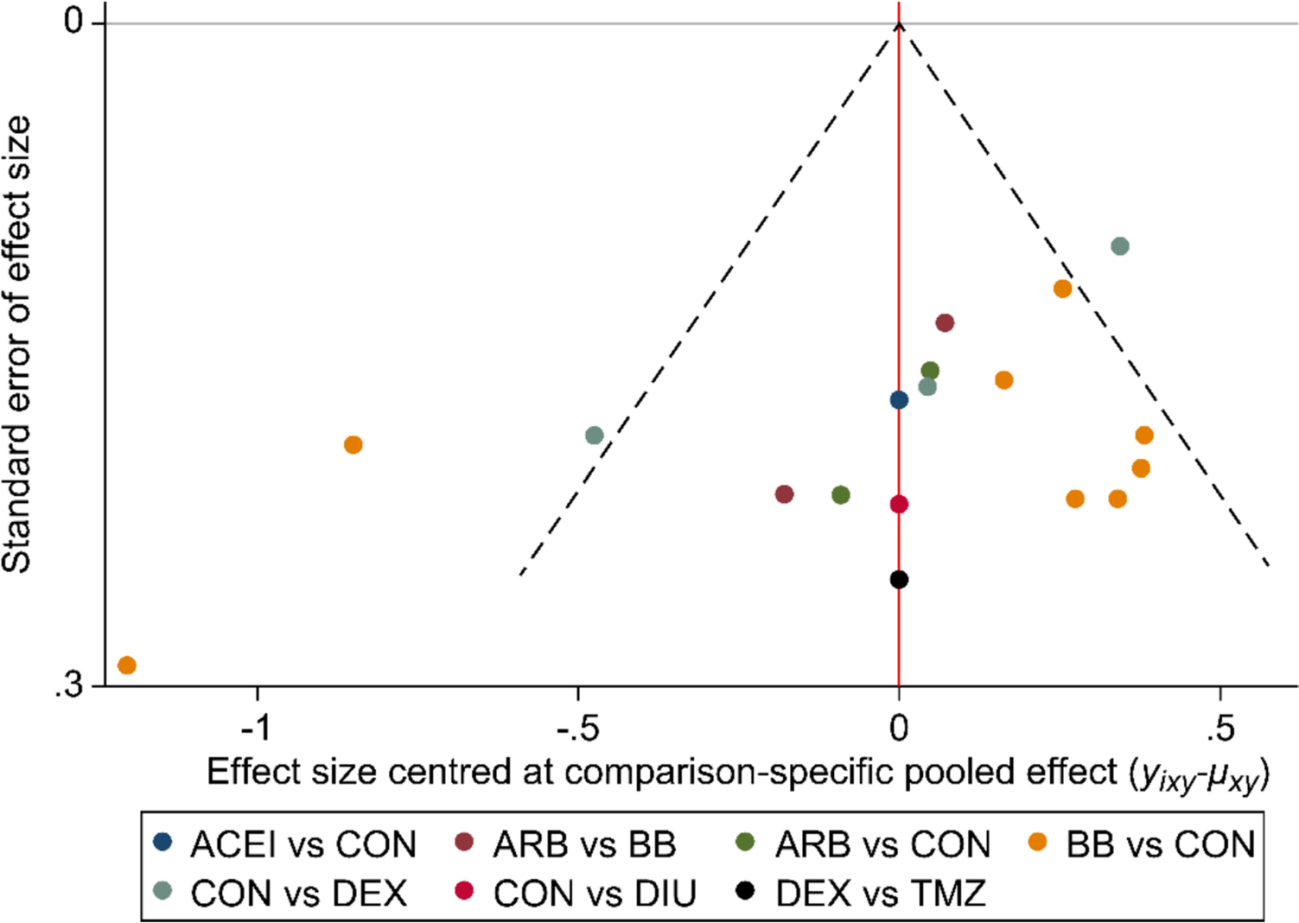
Comparison-Adjusted Funnel Plot for Cardioprotective Agents. The red vertical line denotes the null hypothesis, signifying that individual effect size estimates do not significantly deviate from the pooled estimates for each comparison. Abbreviations: *ACEI* angiotensin-converting enzyme inhibitors, *ARB* angiotensin II receptor blockers, *BB* β1-adrenergic receptor blockers, *MRA* Mineralocorticoid receptor antagonists, *DEX* dexrazoxane, *TMZ* trimetazidine, *CON* control

Figure 4 presents funnel plots assessing publication bias in the primary outcome. A slight indication of publication bias was observed, with one outlier suggesting small-study effects [22]. However, a sensitivity analysis excluding this outlier yielded a symmetrical funnel plot, indicating that small-study effects were unlikely to have significantly influenced the overall findings. The comparison-adjusted funnel plot further evaluated publication bias and heterogeneity among cardioprotective drug studies. While most studies clustered around the pooled effect, asymmetry suggested potential bias or variability, particularly in ARBs and MRAs. In contrast, comparisons involving control versus dexrazoxane, ACEIs, and β-blockers appeared more consistent. The presence of small-study effects suggested some variability in study precision. Despite minor asymmetry, the overall distribution supports the robustness of the meta-analysis, warranting further statistical tests to confirm and adjust for bias.

### Evaluation of certainty of evidence using the GRADE approach

The GRADE assessment (Table 5) evaluated the certainty of evidence for cardioprotective interventions, considering key factors such as risk of bias, inconsistency, imprecision, and indirectness. Comparisons of ARBs, β-blockers, and MRAs against control were classified as moderate certainty evidence, with β-blockers and ARBs consistently maintaining a moderate confidence level.

**Table 5:** Summary of the grade assessment for certainty of evidence

Comparison effect	Number of participants	Number of direct comparisons	Nature of evidence	Certainty	Reason for downgrading
ACEI vs. Control	48 vs. 48	1	Mixed	Very Low	Risk of bias^a^, inconsistency^b^, imprecision^c^
ARB vs. CON	112 vs. 73	2	Mixed	Moderate	Imprecision
BB vs. CON	369 vs. 342	8	Mixed	Moderate	Imprecision
DEX vs. CON	272 vs. 223	3	Mixed	Very Low	Risk of bias^a^, inconsistency^b^, imprecision^c^
DIU vs. CON	43 vs. 40	1	Mixed	Moderate	inconsistency^b^
TMZ vs. CON	0 vs. 0	0	Indirect	Very Low	inconsistency^b^, imprecision^d^
ARB vs. ACEI	0 vs. 0	0	Indirect	Very Low	inconsistency^b^, imprecision^d^
BB vs. ACEI	0 vs. 0	0	Indirect	Very Low	inconsistency^b^, imprecision^d^
DEX vs. ACEI	0 vs. 0	0	Indirect	Very Low	inconsistency^b^, imprecision^d^
DIU vs. ACEI	0 vs. 0	0	Indirect	Very Low	inconsistency^b^, imprecision^d^
TMZ vs. ACEI	0 vs. 0	0	Indirect	Very Low	inconsistency^b^, imprecision^d^
BB vs. ARB	100 vs. 112	2	Mixed	Moderate	imprecision^c^
DEX vs. ARB	0 vs. 0	0	Indirect	Very Low	inconsistency^b^, imprecision^d^
DIU vs. ARB	0 vs. 0	0	Indirect	Very Low	inconsistency^b^, imprecision^d^
TMZ vs. ARB	0 vs. 0	0	Indirect	Very Low	inconsistency^b^, imprecision^d^
DEX vs. BB	0 vs. 0	0	Indirect	Very Low	inconsistency^b^, imprecision^d^
DIU vs. BB	0 vs. 0	0	Indirect	Very Low	inconsistency^b^, imprecision^d^
TMZ vs. BB	0 vs. 0	0	Indirect	Very Low	inconsistency^b^, imprecision^d^
DIU vs. DEX	0 vs. 0	0	Indirect	Very Low	inconsistency^b^, imprecision^d^
TMZ vs. DEX	22 vs. 24	1	Mixed	Very Low	Risk of bias^a^, inconsistency^b^, imprecision^c^
TMZ vs. DIU	0 vs. 0	0	Indirect	Very Low	inconsistency^b^, imprecision^d^

^a^Potential risk of bias due to performance bias. ^b^Potential inconsistency due to not enough data

^c^Confidence intervals encompass values supporting either treatment

^d^Confidence intervals encompass values supporting either treatment; limited sample size

*ACEI* angiotensin-converting enzyme inhibitors, *ARB* = angiotensin II receptor blockers; *BB* β−1 adrenergic receptor blockers; *MRA*, *DEX* dexrazoxane, *TMZ* trimetazidine, *CON* control

However, certain comparisons including ACEIs and dexrazoxane versus control were downgraded to very low certainty. This downgrade was primarily attributed to methodological limitations, substantial heterogeneity, and wide confidence intervals. Indirect comparisons, particularly those without direct supporting studies (e.g., ARB vs. ACEI, dexrazoxane vs. β-blockers, MRAs vs. β-blockers), were further constrained by inconsistency and imprecision.

Direct head-to-head comparisons between cardioprotective agents generally exhibited very low certainty, except for the β-blockers vs. ARBs comparison, which demonstrated moderate certainty based on data from 100 and 112 participants, respectively. The GRADE framework identified significant gaps in current evidence, warranting RCTs to rigorously determine the efficacy and safety of cardioprotective strategies in reducing chemotherapy-induced cardiotoxicity [12].

## Discussion

This NMA constitutes the most comprehensive evaluation of pharmacological interventions aimed at preventing anthracycline-induced cardiotoxicity in patients with breast cancer to date. Cardiotoxicity remains a major contributor to morbidity and mortality among cancer survivors, particularly following treatment with anthracycline-based chemotherapy [39]. The persistent risk of long-term cardiac dysfunction underscores the need for effective cardioprotective interventions. The findings of this analysis suggested that ACEIs, ARBs, β-blockers, trimetazidine, dexrazoxane, and MRAs provide significant cardioprotective effects compared to control treatments. However, the limited availability of direct head-to-head comparisons among these interventions impacts the overall certainty of the evidence. Notably, β-blockers and ARBs were exceptions, demonstrating evidence of moderate certainty. Furthermore, the generalizability of these results is constrained by the predominance of participants with normal LVEF in included studies, limiting their applicability to broader breast cancer populations, particularly those with pre-existing cardiac impairment.

Despite these promising results, substantial between-study heterogeneity was observed, as reflected in the wide predictive intervals across several comparisons, likely attributed to differences in study design, patient populations, chemotherapy regimens, and follow-up durations. Furthermore, indirect comparisons indicated no significant differences between ARBs and β-blockers, whereas MRAs appeared to be more effective than dexrazoxane, suggesting that specific interventions may provide greater benefits in certain patient subgroups.

Additionally, the comparison-adjusted funnel plot revealed potential publication bias, indicating that smaller or negative studies may be underrepresented in the literature. Publication bias can distort treatment rankings, underscoring the need for large-scale RCTs with standardized methodologies. Future research should minimize heterogeneity by implementing standardized patient selection criteria, anthracycline regimens, and cardiotoxicity assessment methods to enhance reliability and reproducibility.

### Safety and secondary outcomes

The secondary outcome analysis provided valuable insights into drug safety profiles and functional outcomes. While most treatments were well tolerated, dexrazoxane was associated with hematologic toxicity, including neutropenia and myelosuppression, whereas ACEIs ***Cardioprotective Efficacy of Pharmacological Interventions.***

Among the evaluated interventions in the NMA, MRAs exhibited the greatest efficacy, demonstrating a significant advantage over control treatments in LVEF in patients with breast cancer receiving anthracycline-based chemotherapy. Similarly, trimetazidine exhibited notable cardioprotective effects, although its role in routine clinical practice remains underexplored [40]. The protective effects of β-blockers and dexrazoxane in preserving LVEF align with the findings from prior observational studies examining the cardioprotective role of β-blockers in mitigating chemotherapy-induced cardiotoxicity [22, 41], contributing further evidence supporting the effectiveness of prophylactic β-blockers and dexrazoxane in preventing LVEF decline during chemotherapy.

Trimetazidine was included in this analysis based on a single small randomized controlled trial [40], and its apparent cardioprotective effect should be interpreted with caution. The limited sample size and absence of independent validation restrict the generalizability of these findings. Moreover, its primary mechanism—enhancing myocardial energy efficiency—does not directly target the established pathways of anthracycline-induced cardiotoxicity, such as oxidative stress and mitochondrial dysfunction. As such, the current evidence remains hypothesis-generating, and robust, mechanistically driven trials are warranted to clarify its potential role in cardio-oncology. In contrast, angiotensin-converting enzyme inhibitors (ACEIs) and angiotensin receptor blockers (ARBs) demonstrated significant cardioprotective effects, aligning with prior evidence supporting their role in mitigating anthracycline-associated cardiac injury [14]. β-blockers and ARBs were among the most extensively studied agents; however, variability in efficacy across trials highlights the need for additional well-powered studies to refine optimal dosing strategies and treatment durations. Continued collaborative efforts in this area are essential to inform evidence-based cardioprotective protocols for patients undergoing anthracycline-based chemotherapy.

Notable findings include the strong protective effects of β-blockers and dexrazoxane, which significantly outperformed control treatments in preserving LVEF and reducing cardiac dysfunction risk. These findings suggest that early initiation of β-blockers, particularly carvedilol and nebivolol, may substantially benefit high-risk patients receiving anthracyclines [6]. Similarly, dexrazoxane remains one of the most effective agents in reducing cardiotoxicity; however, concerns regarding its hematologic toxicity have limited widespread clinical adoption [42].

frequently caused hypotension and dry cough [25, 27]. Nevertheless, β-blockers and ARBs effectively maintained hemodynamic stability and autonomic function, providing additional cardiovascular benefits beyond LVEF preservation [14]. Importantly, DEX significantly reduced heart failure incidence and cardiac adverse events while improving overall survival, reinforcing its role as a key prophylactic strategy despite its hematologic risks [24, 37].

### Strengths and limitations

A key strength of this NMA is the high methodological quality of the included studies, minimizing the risk of bias and enhancing result reliability.

Additionally, this analysis reduces gender-related confounding effects and ensures homogeneity in treatment protocols by focusing exclusively on patients with breast cancer. However, several limitations must be acknowledged. Despite an extensive and comprehensive search strategy, only 15 RCTs fulfilled the eligibility criteria, which may constrain the breadth of available evidence and highlight the need for further high-quality trials in this area. In addition, the relatively small sample sizes and variability in study designs across the included trials may influence the overall strength and certainty of the evidence, potentially limiting the conclusiveness of some comparisons**.** The limited sample sizes in individual studies may contribute to bias, reducing statistical power and generalizability. Moreover, variations in anthracycline dosing, concurrent cancer treatments, and follow-up durations introduce potential confounding factors. Differences in treatment regimens, baseline cardiovascular risk, and cardiotoxicity assessment methods contribute to the observed heterogeneity. Furthermore, publication bias and methodological inconsistencies across RCTs cannot be ruled out, as studies with negative or inconclusive results may be underrepresented. While most cardioprotective interventions demonstrated efficacy, β-blockers, ACEIs, ARBs, and antimitotics did not consistently show protective effects, likely owing to heterogeneity in study populations and unmeasured confounders. Nonetheless, despite these limitations, consistent trends toward cardioprotection—particularly in preserving left ventricular (LV) function—were observed across multiple interventions, reinforcing the clinical relevance of these findings.

### Clinical and research implications

These findings underscored the importance of tailoring cardioprotective strategies based on efficacy, safety profiles, and patient-specific risk factors. The consistent cardioprotective effects demonstrated by angiotensin-converting enzyme inhibitors (ACEIs) and angiotensin receptor blockers (ARBs) reinforce their established role in mitigating anthracycline-induced cardiotoxicity [14]. While β-blockers and ARBs have been extensively investigated, the observed variability in efficacy across trials highlights the necessity for further well-powered studies to elucidate the optimal dosing regimens and treatment durations.

Intriguingly, agents such as MRAs and trimetazidine exhibited notable efficacy, suggesting that metabolic and fluid-regulating interventions may hold a more prominent role in preventing chemotherapy-induced cardiotoxicity than previously recognized [43]. However, the inclusion of trimetazidine in this analysis was based on a single, small randomized controlled trial (RCT) [40]. Despite a seemingly substantial effect size, the limited evidence base and lack of clear mechanistic relevance to anthracycline-induced cardiotoxicity render these specific findings hypothesis-generating. This warrants rigorous validation through robust, independent clinical trials. Furthermore, the overall observed heterogeneity across studies and the potential for publication bias necessitate cautious interpretation, particularly when considering application to populations with pre-existing cardiovascular risk factors [6].

These results, while informative, should be considered largely hypothesis-generating and underscore an urgent need for future well-designed, adequately powered randomized controlled trials (RCTs). Such trials are imperative to validate these preliminary findings, refine optimal treatment rankings, and inform the development of evidence-based clinical guidelines. Future direct head-to-head RCTs are warranted to validate these findings, refine treatment rankings, and optimize cardioprotective strategies for clinical practice. Additionally, future research should explore optimal dosing regimens, combination therapies, and long-term cardiac outcomes to ensure sustained cardioprotection in cancer survivors. Standardized definitions of cardiotoxicity, echocardiographic assessment methods, and long-term follow-up are critical for improving comparability across studies and strengthening the evidence base for clinical decision-making.

## Conclusion

The study findings support the superiority of cardioprotective drugs over control treatments in mitigating anthracycline-induced cardiotoxicity. However, the absence of direct head-to-head comparisons among these cardioprotective interventions in RCTs limits the ability to establish a definitive hierarchical ranking of their therapeutic efficacy.

## Supplementary Information

Below is the link to the electronic supplementary material.

Supplementary file1 (DOCX 92 KB)

## Data Availability

The data utilized in this network meta-analysis were derived exclusively from previously published studies identified through a comprehensive and systematic literature search. All relevant summary data extracted from the included studies are presented in the Supplementary Materials, where applicable, to promote transparency and facilitate reproducibility. No individual participant data or proprietary datasets were used in this analysis. The datasets supporting the findings of this study are available from the corresponding author upon reasonable request. As this study is based solely on published data, ethical approval was not required.
