# Supplementary material for: Pharmacological interventions for anthracycline-induced cardiotoxicity in breast cancer: a systematic review and meta-analysis of randomized controlled trials
Source: Breast Cancer Res Treat. 2025 Aug 5;214(1):1–23. doi: 10.1007/s10549-025-07791-7 (PMC12398477; doi:10.1007/s10549-025-07791-7)
Supplement: Supplementary file 1 — Supplementary file1 (DOCX 92 KB) [file 10549_2025_7791_MOESM1_ESM.docx]

**Supplementary Material**

*Breast Cancer Research and Treatment*

**Pharmacological interventions for anthracycline-induced cardiotoxicity in breast cancer: a systematic review and meta-analysis of randomized controlled trials**

Pinyadapat Vacharanukrauh^1^*, Kyle J. Miller^2^, Sheikh M Alif^1,3^, Fergal Grace^1^, and Muhammad Aziz Rahman^1,4^

^1^Institute of Health and Wellbeing, Federation University Australia, Ballarat, VIC, Australia

^2^School of Psychological Sciences, Monash University, Melbourne, VIC, Australia

^3^School of Public Health and Preventive Medicine, Monash University, Clayton, VIC, Australia.

^4^Faculty of Public Health, Universitas Airlangga, Surabaya, Indonesia

*Corresponding author: Pinyadapat Vacharanukrauh, MD. PhD

Email: [pinyadapatareerob@students.federation.edu.au](mailto:pinyadapatareerob@students.federation.edu.au)

ORCID: 0000-0001-8290-0325

**Inconsistency test between direct and indirect comparisons for cardioprotective effects**

**
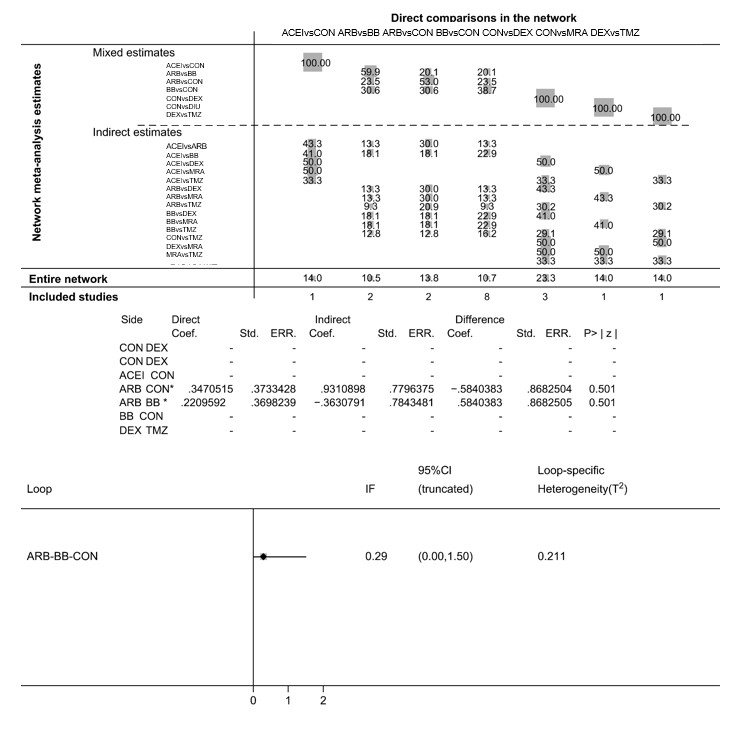
**

MRA

**Fig. S1a** Inconsistency Plot of the Cardioprotective Network with Loop-Specific Heterogeneity Estimates

The plot evaluates inconsistency within the network of cardioprotective agents, considering heterogeneity across treatment loops. Abbreviations: ARB = angiotensin II receptor blockers; BB = β-1 adrenergic receptor blockers; CON = control.

**Fig. S1b** Contribution Plot for the Cardioprotective Network

The plot depicts the relative contribution of direct comparisons to the overall network estimates, where square sizes correspond to the weight of each direct summary effect (horizontal axis) in determining the network summary effect (vertical axis). Abbreviations: ACEI – Angiotensin-Converting Enzyme Inhibitors; ARB – Angiotensin II Receptor Blockers; BB – β1-Adrenergic Receptor Blockers; MRAs – Mineralocorticoid receptor antagonists; DEX – Dexrazoxane; TMZ – Trimetazidine; CON – Control.
